# Like father, like daughter: amoxicillin-induced aseptic meningitis in a female patient and her father: a case report

**DOI:** 10.1186/s13256-026-06579-7

**Published:** 2026-09-18

**Authors:** Ibrahim Farhood, Sabine Seidel, Maximilian Kaeder, Lars Schönlau, Corinna Seliger

**Affiliations:** 1 Department of Neurology, Ruhr University Bochum, Knappschaft Kliniken University Hospital Bochum, In Der Schornau 23-25, 44892 Bochum, Germany; 2 Institute for Diagnostic and Interventional Radiology, Neuroradiology and Nuclear Medicine, Ruhr University Bochum, Knappschaft Kliniken University Hospital Bochum, Bochum, Germany

**Keywords:** Amoxicillin, Meningitis, Hypersensitivity, Headache, Case report

## Abstract

**Background:**

Drug-induced aseptic meningitis (DIAM) is a neurological disorder that requires specialized diagnostic pathways and an in-depth patient history to prevent inaccurate diagnoses and unnecessary procedures. Amoxicillin has been reported as one of the drugs causing recurrent DIAM, with a total of 22 patients described in the literature.

**Case presentation:**

We report two cases involving patients of Mediterranean ethnicity —a father (69-years-old) and his daughter (47-years-old)—who presented to our neurology department at different time points with severe headache persisting for several days. Both patients disclosed recent use of amoxicillin to treat bronchitis and otitis. In both cases, examination of cerebrospinal fluid (CSF) revealed leukocytosis with lymphocytic predominance, while no causative pathogens were identified. After extensive investigations to rule out other causes of secondary headache, a diagnosis of amoxicillin-induced aseptic meningitis (AIAM) was established. Notably, the daughter experienced two similar episodes, both temporally associated with amoxicillin intake.

**Conclusions:**

Amoxicillin-induced aseptic meningitis is a rare adverse reaction that should be considered in patients presenting with severe headache in the absence of an identifiable pathogen. A thorough patient history combined with comprehensive diagnostic evaluation is essential to establish the diagnosis and to avoid unnecessary or excessive treatment. Additionally, potential genetic predispositions, as suggested in other forms of drug-induced aseptic meningitis, may play a role and should be considered in selected cases.

## Background

Aseptic meningitis is characterized by an inflammation of the meninges with a pleocytosis in the cerebrospinal fluid (CSF) without any detectable pathogens. The major causes of aseptic meningitides are systemic diseases, neoplasia, and drug-induced aseptic meningitis (1).

Aseptic meningitis has been reported with an annual incidence of 7.6 cases per 100,000 adults [2]. The true incidence of drug-induced aseptic meningitis (DIAM) remains unknown. A study from the French Pharmacovigilance Database (FPDB) reported 329 cases from (1 January 1985 to 8 March 2017) in France [3]. Nonsteroidal anti-inflammatory drugs (NSAIDs), antibiotics and intravenous immunoglobulins are predominantly implicated as the primary cause of DIAM [1]. Amoxicillin-induced aseptic meningitis has been observed in 22 patients (13 males, 9 females) in a recent Chinese review, exhibiting a predominant prevalence in male patients, although the actual pathophysiology remains unclear [4].

The major clinical symptoms of aseptic meningitis are fever, nuchal rigidity, headache, lethargy, and nausea [5]. We report two new cases that presented to an emergency department of a German University Hospital. Notably, the patients were relatives, which adds an additional aspect to prior observations.

## Case presentation

### Patient 1

A 47-year-old female presented to the neurologic emergency department with a diffuse holocephalic headache radiating to her neck and lower back over the past five days. The patient reported that the headache had started on the second day after initiating amoxicillin therapy for otitis. The pain was accompanied by ocular discomfort, exacerbated by head movements, and significantly impaired concentration. For example, the patient forgot to ensure that her daughter wore a helmet while cycling to school. The patient mentioned discontinuing amoxicillin a few days prior, recalling similar headache episodes 6 and 8 years ago following ear infections treated with amoxicillin. No fever was reported in the recent days. The patient had a medical history of hypothyroidism, migraines, attention deficit hyperactivity disorder ADHD, and bipolar disorder. Two years ago, she experienced a thyroiditis de Quervain. Current medications included L-thyroxin and methylphenidate. Clinical examination revealed increased headache intensity upon passive head and leg movement without fever or meningism. Laboratory results displayed a normal complete blood count with no signs of elevated infection markers. Cerebrospinal fluid (CSF) examination on the day of presentation revealed a mild pleocytosis (19 cells/μL, normal range 0–4 cells/μL) with normal glucose, lactate, and protein (Table 1). The PCR for neurotropic pathogens (Escherichia coli K1, Haemophilus influenzae, Listeria monocytogenes, Neisseria meningitidis, Streptococcus agalactiae, Streptococcus pneumoniae, Cytomegalievirus, Enterovirus, Herpes simplex Virus 1, Herpes simplex Virus 2, Human Herpesvirus 6, Human Parechovirus, Varizella Zoster Virus, Cryptococcus neoformans/gattii) yielded negative results. CSF cultures were also performed and remained sterile. No empirical antimicrobial therapy was administered before lumbar puncture.

**Table 1:** CSF analysis in patient 1

Parameter	Normal range	Unit	Result
Protein	20.0—50.0	mg/dl	36.70
Glucose	30—80	mg/dl	65.10
Lactat	10.0—20.0	mg/dl	14.45
Oligoclonal bands	Negative		Negative
Leucocytes	< 4	/µl	19
Erythrocytes	0	10^3/µl	0.00
Mononuclear cells		/µl	19.00
Mononuclear cells		%	100
Polymorphonuclear neutrophil leukocytes**)**		/µl	00.00
Polymorphonuclear neutrophil leukocytes		%	00.00

Further examination of the patient’s history revealed two another episodes with a similar CSF profile occurring after Amoxicillin intake 6 and 8 years ago, consistent with episodes of aseptic meningitis following its administration. The brain MRI exhibited nonspecific white matter lesions categorized as microangiopathy. Suspecting a chronological association between Amoxicillin and meningitis, and considering documented cases in the literature, we initiated treatment with paracetamol, resulting in sufficient pain relief. The patient was discharged six days after treatment initiation.

### Patient 2

The father of the aforementioned patient, a 69-year-old man, presented to our emergency department several years after the discharge of his daughter. He complained of a severe holocephalic headache, with emphasis on the right frontal area, over the past three days. The patient reported that the headache had started on the second day after initiating amoxicillin therapy for bronchitis. Notably, there was no trigeminal involvement, and the patient had no prior history of headaches. Despite taking paracetamol in the preceding days, there was no significant pain relief. The patient had been receiving amoxicillin therapy for bronchitis for four days before presentation. He had a history of a cardiac attack a few years ago, type two diabetes with hyperuricemia, and was regularly taking ASS, atorvastatin, bisoprolol, allopurinol, metformin and sitagliptin, No fever was reported in the recent days. Clinical examination revealed only hyporeflexia in the lower extremities.

**Fig. 1:**
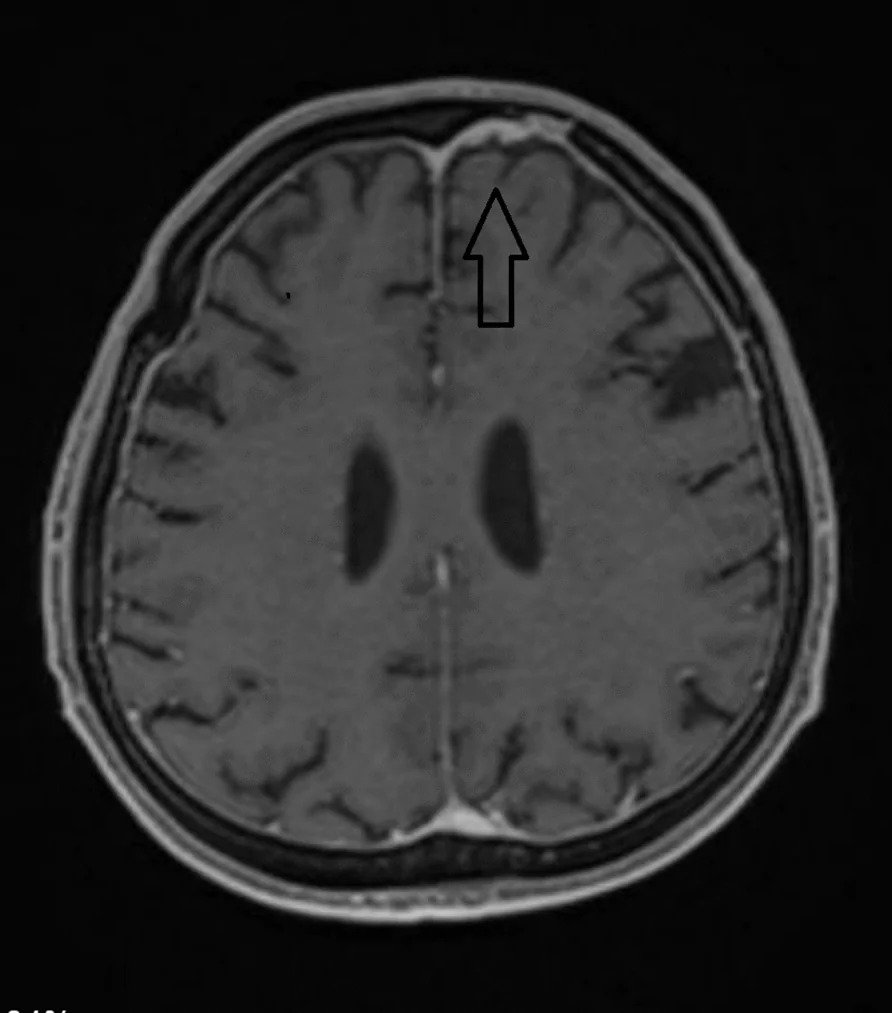
Brain MRI of Patient 2 showing thickening and enhancement of the dura mater with contrast medium enhancement (arrow)

Further investigations were initiated, including an MRI that demonstrated thickening and enhancement of the dura mater with contrast medium enhancement (Fig. 1). CSF analysis on the day of presentation revealed 117 cells/μL (normal range 0–4 cells/ul) with elevated CSF protein levels (93 mg/dL, normal range 20–50 mg/dl), indicative of a lymphocytosis, and slightly elevated lactate levels (21 mg/dl, normal range 10–20 mg/dl), while glucose levels remained within the normal range (Table 2). Empirical antimicrobial therapy with intravenous ceftriaxone (initially 2 g every 12 h, subsequently reduced to 2 g once daily), ampicillin (2 g every 4 h), and aciclovir (10 mg/kg every 8 h) was initiated after cerebrospinal fluid sampling. Antimicrobial therapy was discontinued after two days when CSF cultures and multiplex PCR remained negative and an infectious etiology was excluded through Multiplex-PCR. CSF cultures remained negative.

**Table 2:** First CSF analysis in patient 2

Parameter	Normal range	Unit	Result
Protein	20.0—50.0	mg/dl	93.70 +
Glucose	30—80	mg/dl	75.10
Lactat	10.0—20.0	mg/dl	21.51 +
Oligoclonal bands	Negative		negative
Leucocytes	< 4	/µl	117.0 +
Erythrocytes	0	10^3/µl	0.00
Mononuclear cells		/µl	117.00
Mononuclear cells		%	100.00
Polymorphonuclear neutrophil leukocytes**)**		/µl	0.00
Polymorphonuclear neutrophil leukocytes		%	0.00

Additional laboratory findings showed a slightly elevated ANA-Titer. CRP was not elevated. Considering Amoxicillin as the cause of meningitis, a decision was made to adopt a watchful waiting approach. After 5 days, a second lumbar puncture revealed a decrease in pleocytosis to 62 cells/μL, with normal lactate, protein, and glucose levels (Table 3). This improvement coincided with a reduction in pain, leading to the patient's discharge on day 6.

**Table 3:** Second CSF analysis in patient 2

Parameter	Normal range	Unit	Result
Protein	20.0—50.0	mg/dl	46.00
Glucose	30—80	mg/dl	58.50
Lactat	10.0—20.0	mg/dl	18.72
Oligoclonal bands	Negative		negative
Leucocytes	< 4	/µl	62.0 +
Erythrocytes	0	10^3/µl	0.00
Mononuclear cells		/µl	61.00
Mononuclear cells		%	98.40
Polymorphonuclear neutrophil leukocytes**)**		/µl	1.00
Polymorphonuclear neutrophil leukocytes		%	1.60

## Discussion and conclusions

Although the true worldwide incidence of drug-induced aseptic meningitis (DIAM) remains unknown, several large reviews and pharmacovigilance studies provide an overview of the reported cases and the drugs most frequently implicated. The French National Pharmacovigilance Database identified 329 DIAM cases, with intravenous immunoglobulins (30%), NSAIDs (14%), antimicrobials (11%), monoclonal antibodies (8%), and vaccines (7%) representing the leading drug classes. Garcia-Monco et al. summarized 194 episodes, in which NSAIDs (37%) and antibiotics (36%) predominated; ibuprofen (23.7%), trimethoprim–sulfamethoxazole (16.5%), lamotrigine (15.5%), and amoxicillin (4.1%) were the most frequently reported individual agents [3]. Similar findings were reported by Kalmi et al. (151 cases) [6], while the recent systematic review by Singh et al. (108 patients) confirmed ibuprofen (24%), trimethoprim–sulfamethoxazole (14%), naproxen (11%), amoxicillin/clavulanate (9%), infliximab (7%), nivolumab (6%), and intravenous immunoglobulin (6%) among the most commonly implicated drugs [7]. These data are summarized in Table 4.

**Table 4:** Published studies summarizing drug-induced aseptic meningitis (DIAM) according to causative drug classes and the most frequently implicated individual agents

Study (Year)	Study design	DIAM cases (n)	Most frequent drug classes	Most frequent individual drugs
Garcia-Monco et al., 2014	Literature aggregation (192 publications)	194 episodes	NSAIDs 72 (37%), antibiotics 69 (36%), immunosuppressive/immunomodulatory drugs 19 (10%), antiepileptics 34 (18%)	Ibuprofen 46 (23.7%), TMP–SMX 32 (16.5%), Lamotrigine 30 (15.5%), Amoxicillin 8 (4.1%)
Kalmi et al., 2020	Literature review (1995–2017)	151 cases	NSAIDs 49 (32.5%), antibiotics 46 (30.5%), biologics 19 (12.6%), immunomodulators 15 (9.9%), other drugs 22 (14.6%)	Ibuprofen, Naproxen, Sulindac, TMP–SMX, Trimethoprim, Amoxicillin ± clavulanate, Cetuximab, Infliximab, Adalimumab, Efalizumab, Lamotrigine, Carbamazepine, Methotrexate, Sulfasalazine, Leflunomide, IVIG, Allopurinol, Nevirapine, Pemetrexed, Cytarabine, contrast media (no pooled frequencies reported)
Bihan et al., 2019	National pharmacovigilance study (French Pharmacovigilance Database, 1985–2017)	329 cases (203 well documented)	IVIG 30%, NSAIDs 14%, antimicrobials 11%, monoclonal antibodies 8%, vaccines 7%	Frequently implicated drugs included IVIG preparations, Ibuprofen, Amoxicillin, TNF inhibitors (Infliximab, Adalimumab, Etanercept), and intrathecal Methotrexate/Cytarabine. Individual drug frequencies were not reported
Singh et al., 2026	Systematic review of case reports and case series (25 years)	108 patients	In adults: NSAIDs 30/93 (32%), monoclonal antibodies 22/93 (24%); antibiotics were the second largest drug class overall	Ibuprofen 26 (24%), TMP–SMX 15 (14%), Naproxen 12 (11%), Amoxicillin/clavulanate 10 (9%), Infliximab 8 (7%), Nivolumab 7 (6%), IVIG 7 (6%), Ketorolac 5 (5%), Cetuximab 4 (4%), Isotretinoin 4 (4%)

TMP–SMX = trimethoprim–sulfamethoxazole; IVIG = intravenous immunoglobulin; NSAIDs = non-steroidal anti-inflammatory drugs

As a beta-lactam antibiotic, amoxicillin is the most frequently prescribed antibiotic worldwide. Amoxicillin-induced aseptic meningitis (AIAM) is considered a rare disorder that has been reported in 22 patients according to the latest review. In those few reported cases, men are more likely to develop this disorder [4]. The men/women predominance differs depending on the drug itself, since Trimethoprim-Sulfamethoxazole and intravenous immunoglobulin are observed more in women [8, 9].

This disorder may occur at any age, even in children, and might be recurrent [9].

Most patients present with headache, fever, nuchal rigidity, nausea, and vomiting [1]. To prevent misdiagnosis, it is necessary to perform a careful investigation with a comprehensive patient history followed by a lumbar puncture. Spontaneous recovery is common, and symptomatic therapy, including pain relievers such as paracetamol, proves effective [9].

The pathophysiology of aseptic meningitis is still unclear. Some theories suggest the irritation of meninges by drugs circulating in the cerebrospinal fluid. This irritation may be due to their chemical structures, such as lipid solubility, pH, and size [10]. If this theory is believed to be the cause of aseptic meningitis, then we need to prove the ability of each drug to circulate from the blood to the central nervous system. On the other hand, an immunological theory might be behind aseptic meningitis. This theory might be plausible since there is a correlation between exposure to certain drugs and induced allergic reactions. The prevailing scientific perspective attributes this phenomenon to a delayed type IV hypersensitivity reaction, characterized by T-cell-mediated hypersensitivity [11]. This process starts with the sensitization phase, where the antigen interacts with T cells, leading to their activation and expansion. This phase is followed by the effector phase when a new exposure to the antigen occurs, which leads to cytokine secretion such as interleukin-2 (IL-2) and interferon-γ (IFN-y). This type of reaction emerges 24–48 h after prior exposure to the drug [12].

The reported cases represent the first instances observed in relatives, with no predisposition for this disorder mentioned thus far. Due to the rarity of amoxicillin-Induced Aseptic Meningitis (AIAM), it is unclear whether its manifestation in relatives is a coincidence or if there might be some environmental or genetic predisposing factors. However, considering type IV hypersensitivity in the pathogenesis of aseptic meningitis, we may find a possible genetic predisposition explaining our observation. The current theories on drug hypersensitivity suggest an involvement of the Human Leukocyte Antigen (HLA). For instance, the hapten concept suggests binding of a drug or its metabolites ('hapten') to a large protein, which is processed in the antigen-presenting cells and presented to T cells on HLA molecules to induce T cell activity [13]. The pharmacological interactions concept as a second theory suggests the activation of T cells with hypersensitivity through direct binding with the T cells or HLA molecules [14]. The Altered HLA Peptide Repertoire-Binding concept indicates as a third theory the binding of drugs to HLA molecules, leading to changes in the HLA peptide repertoire. This repertoire is then considered strange for T cells and leads to T cell expansion and hypersensitivity [15]. All those theories about the role of HLA in drug hypersensitivity reactions led researchers to look for specific HLA alleles in some drug hypersensitivity reactions. For instance, HLA-B57:01 was found to be associated with hypersensitivity against abacavir [16], whereas carbamazepine hypersensitivity was associated with HLA-B15:02 and HLA-A31:01 [17, 18], and flucloxacillin with HLA-B57:01 [19]. A specific HLA association with amoxicillin-induced aseptic meningitis might be a possible explanation for this first observation in our patient and her father. However, AIAM is rare and only observed in a limited number of cases in few patients, which makes the association to certain HLA alleles challenging. By documenting these cases, our intention is to contribute to a more profound understanding of the disease and facilitate further research in this field. There are some limitations associated with this case study. One limitation is that there were very few cases studied, and thus the results cannot be generalized to all other similar cases. Another limitation is that no genetic tests were conducted to determine any hereditary predisposition.

The present case report has several limitations. First, the exact commercial brand and formulation of amoxicillin used by both patients could not be determined retrospectively. Second, HLA typing or other genetic analyses were not performed; therefore, the hypothesis of a genetic predisposition remains speculative. Third, as this report describes only two related patients, no causal conclusions regarding familial susceptibility can be drawn. Finally, although the temporal relationship, recurrence after amoxicillin exposure, and exclusion of infectious causes strongly support the diagnosis of amoxicillin-induced aseptic meningitis, confirmation by drug rechallenge was not performed because of ethical and safety considerations.

To sum up, AIAM is a rare condition that usually manifests symptoms like headache, fever, and neck stiffness. It is crucial to conduct a detailed patient history and thorough diagnostic assessment to identify this condition accurately. The probable mechanism behind this condition is believed to involve a type IV hypersensitivity response.

## Data Availability

No datasets were generated or analysed during the current study.

## Abbreviations

AIAM: Amoxicillin-induced aseptic meningitis
ANA: Antinuclear antibodies
ASS: Acetylsalicylic acid
CRP: C-reactive protein
CSF: Cerebrospinal fluid
DIAM: Drug-induced aseptic meningitis
FPDB: French pharmacovigilance database
HLA: Human leukocyte antigen
HSV-1/HSV-2: Herpes simplex virus type 1 / 2
IFN-γ: Interferon gamma
IL-2: Interleukin 2
MRI: Magnetic resonance imaging
NSAIDs: Nonsteroidal anti-inflammatory drugs
PCR: Polymerase chain reaction
